# Encapsulation of Pomegranate Polyphenols in Plant-Based Proteins and Bioactivity of Resulting Microparticles

**DOI:** 10.3390/foods15122189

**Published:** 2026-06-17

**Authors:** Mirela Kopjar, Mary Ann Lila, Anureet K. Thind, Josip Šimunović, Dražen Raucher

**Affiliations:** 1 Faculty of Food Technology Osijek, Josip Juraj Strossmayer University in Osijek, Franje Kuhača 18, 31000 Osijek, Croatia; 2 Department of Food, Bioprocessing and Nutrition Science, Plants for Human Health Institute, North Carolina State University, North Carolina Research Campus, 600 Laureate Way, Kannapolis, NC 28081, USA; mlila@ncsu.edu; 3 Department of Cell and Molecular Biology, University of Mississippi Medical Center, 2500 North State Street, Jackson, MS 39216, USA; athind@umc.edu; 4 Department of Food, Bioprocessing and Nutrition Science, North Carolina State University, 116C Schaub Hall, Raleigh, NC 27695, USA; simun@ncsu.edu

**Keywords:** polyphenols, pomegranate juice, pea proteins, rice proteins, bioactivity

## Abstract

The main objective of this study was to generate protein-polyphenol microparticles on the basis of pea and rice proteins in combination with pomegranate juice. Protein microparticles were prepared as a freeze-dried powder and evaluated for total polyphenols and proanthocyanidins using spectrophotometric methods, and for individual polyphenols using the HPLC method. In addition, they were assessed for antioxidant activity, and IR spectra were recorded to establish structural changes in proteins upon adsorption of pomegranate polyphenols. The potential of the formulated microparticles to inhibit colon cancer cell proliferation (SW1116 and Colo205) was also investigated and compared with pomegranate juice. The adsorption capacity of total polyphenols for both protein matrices were 47%. All compounds had a higher affinity for the pea protein matrix except gallic acid. The highest affinity for proteins had punicalagin, with 88% and 80% for pea and rice proteins, respectively. The microparticles demonstrated antioxidant potential using DPPH, ABTS, FRAP, and CUPRAC methods. Both pomegranate juice and protein microparticles exhibited high antioxidant potential and inhibitory effects on both types of colon cancer cells. Screening of IR spectra of protein microparticles revealed the adsorption of pomegranate polyphenols through changes in protein structures, particularly in regions characteristic of proteins.

## 1. Introduction

Over the years, the food industry has become more concentrated and focused on developing innovative products with potential health benefits, such as antioxidant function, antidiabetic activity, anti-inflammatory action, and anti-cancer properties [1,2]. A modern and hectic lifestyle has a significant influence on consumers’ dietary habits, which, over time, can result in negative health consequences. Nutritional guidelines and recommendations emphasize the significance of the intake of fruits and their products. To overcome these obstacles, encapsulation is used nowadays; however, its success depends on selecting appropriate biopolymers that can bind most polyphenols from selected sources and largely preserve their activity. Proteins are among the most studied biopolymers, with an emphasis on plant-based proteins. The use of plant-based proteins is increasing, and it is estimated that by 2030, 7.7% of the global protein market will consist of plant-based foods, resulting in a market value of over US$162 billion, compared to US$29.4 billion in 2020. This strong interest in plant-based proteins is linked to a noticeable increase in population, greater environmental awareness, improved consumer understanding of healthy food choices, and the importance of sustainability [3]. Consequently, studies on plant-based proteins have expanded over the years, aiming to utilize them as functional food ingredients, either alone or in combination with other compounds, especially bioactives. Among bioactives, considerable focus has been directed towards polyphenols. Plant proteins are used in food products due to various technological and functional properties, as well as their ability to encapsulate polyphenols. Many studies have been conducted with this aim, using different types of proteins and various sources of polyphenols, highlighting the chemical structures and properties of proteins and polyphenols as the main factors influencing encapsulation efficiency [4,5,6,7,8,9,10,11,12,13,14,15].

In the Mediterranean, pomegranate (*Punica granatum L.*) is a native fruit; however, cultivation and consumption have spread worldwide [16]. In folk medicine, it is known for its positive impact on human health, through antioxidant, antimicrobial, antidiabetic, anticancer, and anti-inflammatory effects [17]. These effects are related to its high content of bioactive compounds, including anthocyanins, phenolic acids, flavonoids, proanthocyanidins, lignin, phytosterols, organic acids, and fatty acids [18]. Pomegranate is a rich source of punicalagin, a tannin that can be hydrolyzed and whose chemical structure consists of multiple esters of gallic acid and ellagic acid with glucose [16]. The high popularity of this fruit worldwide and its potential health benefits have led to its increased cultivation and the production of various products [19]. Due to the recognized health benefits of pomegranate juice and its components, it is necessary to protect them and ensure their stability. Because polyphenols are known to be unstable under various conditions, converting them into a dried form or other encapsulated forms is a possible way to address these issues. Most studies on the encapsulation of pomegranate polyphenols have focused on encapsulating pomegranate peel extract using various methods, primarily with carbohydrates as carriers, as summarized in the review by Rahul et al. [20]. Maltodextrin or soybean protein isolates have been used to encapsulate pomegranate juice and extracts by spray drying [21], while extracts have also been encapsulated by ionic gelation using pectin alone or with added starch [22]. The use of plant-based proteins for encapsulating pomegranate juice polyphenols is a poorly investigated area; therefore, we selected rice and pea protein matrices. Both proteins have gained attention in recent years, especially due to their hypoallergenic properties compared to mainstream soy protein [23,24,25,26]. Additionally, rice proteins are valued for their digestibility and balanced amino acid composition [23,24]. These attributes make them ideal replacements for dairy and soy proteins in various applications, including nutritional beverages, infant foods, and other protein-enriched foods. Pea proteins are valued not only for their high quality but also for their abundance and low production costs. Like rice proteins, they have a good amino acid profile and can meet human nutritional needs. Pea proteins also maintain better structural integrity at higher temperatures, making them more suitable for use in foods processed under different conditions [25,26]. This also contributes to the expansion of the field of developing new or improved functional additives.

Consequently, the protein microparticles were prepared by complexing pea and rice proteins with pomegranate juice to expand knowledge in the encapsulation of pomegranate polyphenols on plant-based proteins, more precisely rice and pea proteins. The bioactivity of the resulting protein microparticles was determined through evaluation of total polyphenols, proanthocyanidins, individual polyphenols, antioxidant activity, and inhibition of colon cancer cells. Additionally, IR spectra were recorded to confirm the adsorption of polyphenols on proteins. Additionally, the behavior, i.e., affinity, of pomegranate juice polyphenols toward rice and pea proteins was compared.

## 2. Materials and Methods

### 2.1. Compounds for the Generation of Protein Microparticles and Chemicals for Analysis

Pomegranate juice was prepared by peeling the fruit and separating the seeds, which were then used to obtain the juice by pressing. Firstly, the mixture, after pressing, was filtered and heated at 90 °C for 3 min to inactivate enzymes and vegetative microorganisms, achieving a stable system for continued use. Protein microparticles were generated using two protein matrices as carriers, the pea protein matrix (Blesterfeld, Hamburg, Germany) and the rice protein matrix (Biovega, Zagreb, Croatia).

Solvents for extraction and spectrophotometric analysis, hydrochloric acid (37%), and methanol were supplied by Carlo Erba Reagents (Sabadell, Spain), while acetic acid (>99.5%) was from Alkaloid (Skopje, North Macedonia) and ethanol from Gram-mol (Zagreb, Croatia). Ammonium acetate, sodium acetate, calcium chloride, and potassium chloride were sourced from Gram-mol (Zagreb, Croatia). Sodium carbonate was obtained from T.T.T. (Sveta Nedelja, Croatia), while the Folin–Ciocalteu reagent and potassium persulfate were sourced from Kemika (Zagreb, Croatia). Neocuproine (>99%), 2,4,6-tri(2-pyridyl)-s-triazine (99%), and cupric chloride were supplied by Acros Organic (Geel, Belgium). 2,2′-azino-bis(3-ethylbenzothiazoline-6-sulphonic acid) diammonium salt (>98%), 2,2-diphenyl-1-picrylhydrazyl, 4-dimethylaminocinnamaldehyde, and Trolox (97%) were obtained from Sigma-Aldrich (St. Louis, MO, USA). Some standards, such as procyanidin B2 (>90%) and punicalagin (>98%), were also sourced from Sigma-Aldrich (St. Louis, MO, USA), while standards of cyanidin-3-glucoside (>96%), delphinidin-3-glucoside, gallic acid (>97%), and ellagic acid (>95%) were sourced from Extrasynthese (Genay, France). The two mobile phases used for HPLC analysis (both HPLC grade), i.e., orthophosphoric acid and methanol, were sourced from Fisher Scientific (Loughborough, UK) and J.T. Baker (Deventer, The Netherlands), respectively.

To test the inhibition of colon cancer cells, two cell lines, SW1116 and Colo205, were used, both obtained from ATCC (Manassas, VA, USA). Their cultivation was conducted in RPMI 1640 medium (ATCC modification) with the addition of 10% fetal bovine serum (FBS, Gibco, Thermo Fisher Scientific, Waltham, MA, USA) and 1% penicillin–streptomycin solution (Gibco, Thermo Fisher Scientific, USA). Cells were held in a humidified incubator at 37 °C with 5% CO_2_. They were subsequently passaged using a 0.25% trypsin–EDTA solution (Gibco, Thermo Fisher Scientific, USA) and washed with phosphate-buffered saline (PBS, pH 7.4, Gibco, Thermo Fisher Scientific, USA) prior to experiments. Cell proliferation and viability were assessed using the Cell Counting Kit obtained from Dojindo Laboratories (Kumamoto, Japan). The 5-fluorouracil was sourced from Sigma-Aldrich (St. Louis, MO, USA). Analytical grade reagents were used.

### 2.2. Generation of Protein Microparticles

Protein microparticles were prepared by complexing 10% of the protein matrix (pea or rice protein matrix, each containing 85% of proteins) with 20 mL of pomegranate juice. Complexation was carried out on a magnetic stirrer during mixing for 20 min at room temperature. Upon complexation, the mixture was centrifuged for 15 min at 8000 rpm, followed by the separation of the supernatant from the sediment. The sediment was freeze-dried to gain the complexes in powder form. Freeze-drying was carried out in a laboratory freeze-drier Alpha 1–4 (Martin Christ, Osterode am Harz, Germany). Requirements for the freeze-drying were as follows: the freezing temperature was adjusted to −55 °C, the sublimation temperature was adjusted from −35 to 0 °C under a vacuum of 0.220 mbar, and the isothermal desorption temperature was adjusted from 0 to 22 °C under a vacuum of 0.080 mbar. Prior to freeze-drying, the sediments were frozen for 24 h at −18 °C. The entire process lasted 5 h.

### 2.3. Extraction of Protein Microparticles

Two hundred milligrams of microparticles were blended with 1 mL of acidified distilled water (pH 3.5). The entire mixture was incubated for 3 h with constant stirring at 37 °C. After incubation, the mixture was centrifuged to obtain a clear extract, which was used for all further analyses.

### 2.4. Determination of Total Polyphenols

The amount of total polyphenols was determined using the Folin–Ciocalteu (F-C) method as previously defined by Singleton and Rossi [27]. In brief, 0.1 mL of extract, 0.9 mL of distilled water, 5 mL of F-C reagent (3.3%), and 4 mL of sodium carbonate (7.5%) were added to a test tube. The mixture was homogenized and left in the dark at room temperature for 2 h. The absorbance was scanned at 765 nm. Analyses were carried out in triplicate, and the data were presented as mg of gallic acid per g of sample (mg GAE/g).

### 2.5. Determination of Proanthocyanidins

The amount of proanthocyanidins was determined using the 4-(dimethylamino)cinnamaldehyde (DMAC) method as previously defined by Prior et al. [28]. Briefly, 0.5 mL of extract and 1 mL of DMAC reagent were added to a test tube, and the mixture was homogenized and left in the dark at room temperature for 30 min. The absorbance was scanned at 640 nm. The analyses were carried out in triplicate, and the data were presented as mg of proanthocyanidin B2 per g of sample (mg PB2E/g).

### 2.6. Identification and Quantification of Polyphenols

High-performance liquid chromatography (HPLC) was utilized to evaluate specific polyphenols. The 1260 Infinity II HPLC system (Agilent Technologies, Santa Clara, CA, USA) was used, consisting of a diode array detector (DAD) and a quaternary pump. Ten microliters of filtered extract, passed across a PTFE filter (0.45 µm), were injected into the HPLC system, which included a Poroshell 120 column (EC C-18, 4.6 × 100 mm, 2.7 µm) under the flow rate of 1 mL/min. The mobile phases used for elution were phase A: 0.1% H_3_PO_4_ in water and phase B: 100% methanol. The following gradient elution was applied: 0 min, 5% B; 3 min, 30% B; 15 min, 35% B; 22 min, 37% B; 30 min, 41% B; 32 min, 45% B; 40 min, 49% B; 45 min, 80% B; 48 min, 80% B; 50 min, 5% B; and 53 min, 5% B. UV-Vis spectra were scanned in the range of 190 nm to 600 nm. Anthocyanins were measured at 520 nm, phenolic acids at 280 nm, and punicalagin at 260 nm. Standards were used to identify polyphenols; sample chromatograms were compared with standards for both retention times and UV-Vis spectra. For each calibration curve, the stock solutions of the polyphenol standard were prepared, and six concentrations of each standard were used for the construction of calibration curves. Calibration curves were prepared for cyanidin-3-glucoside (concentration range from 1 to 150 mg/L; r^2^ = 0.9999; LOD = 0.029 mg/L; LOQ = 0.085 mg/L), delphinidin-3-glucoside (concentration range from 3 to 150 mg/L; r^2^ = 0.9998; LOD = 0.045 mg/L; LOQ = 0.138 mg/L), gallic acid (concentration range from 1 to 300 mg/L; r^2^ = 0.9998; LOD = 0.160 mg/L; LOQ = 0.390 mg/L), ellagic acid (concentration range from 1 to 150 mg/L; r^2^ = 0.9993; LOD = 0.086 mg/L; LOQ = 0.180 mg/L) and punicalagin (concentration range from 1 to 150 mg/L; r^2^ = 1; LOD = 0.813 mg/L; LOQ = 2.465 mg/L). Cyanidin-3,5-diglucoside and delphinidin-3,5-diglucoside were quantified using the calibration curves of cyanidin-3-glucoside and delphinidin-3-glucoside, respectively. Analyses were carried out in duplicate, and data were presented as mg of the compound per kg of sample (mg/kg).

### 2.7. Determination of Adsorption Capacity

This parameter [29] was determined for total polyphenols, proanthocyanidins, and all individual polyphenols using the following equation:

(1) AdC (%) = ((Jc − Sc)/Jc) × 100

where AdC is the adsorption capacity, Jc is the concentration of previously mentioned compounds in the pomegranate juice, and Sc is the concentration of previously mentioned compounds in the supernatant.

### 2.8. Determination of Antioxidant Potential

The antioxidant protocols used to estimate antioxidant potential were DPPH, ABTS, FRAP, and CUPRAC. Sample absorbance was scanned using a UV-Vis spectrophotometer (Cary 60, Agilent Technologies, Santa Clara, CA, USA). Analyses were carried out in triplicate, with data presented as μmol of Trolox equivalents per g of sample (μmol TE/g). All protocols were previously defined as follows: the DPPH assay by Brand-Williams et al. [30], the ABTS assay by Arnao et al. [31], the ferric reducing power (FRAP) assay by Benzie and Strain [32], and the cupric reducing antioxidant capacity (CUPRAC) assay by Apak et al. [33].

### 2.9. Inhibition of Colon Cancer Cells by Protein Microparticles

Two types of colon cancer cells, SW1116 and Colo205, were selected. They were cultivated in microplates for 24 h, after which the cells were treated with 1%, 3%, and 5% of pomegranate juice or extracts of protein microparticles. Juice/extracts were prepared in specific media: RPMI 1640 medium (ATCC modification with the addition of 10% fetal bovine serum and 1% penicillin/streptomycin). For the negative control, cancer cells were not treated with juice/extracts, while for the positive control, they were treated with 5-fluorouracil (a drug used for colon cancer treatment). After 72 h of treatment, the survival of cancer cells was determined by application of the CCK-8 method. This method measures the activity of dehydrogenase enzymes in metabolically active cells. These enzymes reduce 2-(2-methoxy-4-nitrophenyl)-3-(4-nitrophenyl)-5-(2,4-disulfophenyl)-2H-tetrazolium, monosodium salt (WST-8) to a colored formazan product, which is quantified by absorbance at 450 nm.

The percentage of surviving cancer cells was calculated according to the following formula:

(2) % of survival of cancer cells = (A_treated cells_/A_u__ntreated cells_) × 100

where A_treated cells_ is the absorbance at 450 nm of samples where cancer cells were treated with the pomegranate juice or extracts, and A_untreated cells_ is the absorbance at 450 nm of samples where cancer cells were not treated with the pomegranate juice or extracts.

### 2.10. Recoding of IR Spectra

The protein matrix and protein microparticles were used for recording IR spectra by Fourier transform infrared spectroscopy–attenuated total reflectance (FTIR-ATR) analysis. The FTIR-ATR instrument (Cary 630; Agilent, Santa Clara, CA, USA) contained MicroLab Expert 1.3 (Agilent, Santa Clara, CA, USA) software for spectral analysis. The recording of IR spectra was carried out in the interval from 4000 to 600 cm^−1^. Samples were placed directly on the ATR crystal and pressed against it, and then scanned to obtain IR spectra at a resolution of 4 cm^−1^ and at room temperature, collecting 32 scans.

### 2.11. Statistical Analysis

Investigation data are presented as mean value ± standard deviation (SD). They were statistically analyzed by Statistica 13.1 (StatSoft, Tulsa, OK, USA) by applying one-way ANOVA and Fisher’s LSD test.

## 3. Results

### 3.1. Polyphenols and Antioxidant Potential of Protein Microparticles

**Figure 1:**
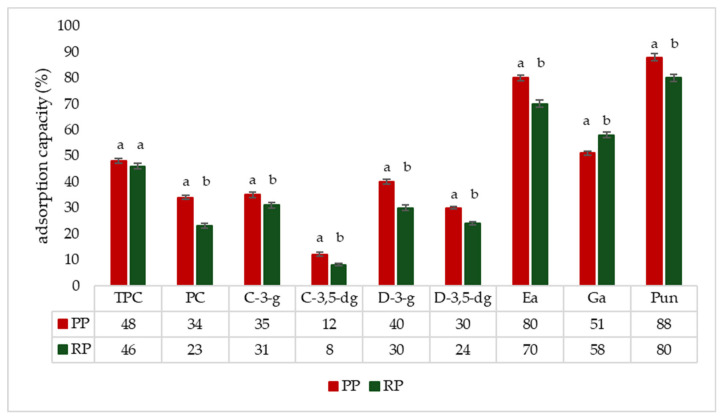
Adsorption capacity of polyphenols on protein matrices (PP—pea protein; RP—rice protein; TPC—total polyphenols; PC—proanthocyanidins; C-3-g—cyanidin-3-glucoside; C-3,5-dg—cyanidin-3,5-diglucoside; D-3-g—delphinidin-3-glucoside; D-3,5-dg—delphinidin-3,5-diglucoside; Ea—ellagic acid; Ga—gallic acid; Pun—punicalagin). Values a and b represent statistically different results by ANOVA and Fisher’s (LSD) test with *p* < 0.05.

Results of the adsorption capacities of polyphenols on protein matrices are presented in Figure 1. By comparing the affinity of both protein matrices towards the adsorption of pomegranate polyphenols, it can be seen that the adsorption capacity for total polyphenols was around 47%. However, for specific polyphenols, these values differ depending on the type of proteins and polyphenols.

All compounds had a higher affinity for pea protein except gallic acid. Regarding proanthocyanidins (PC), their adsorption capacities were 34% and 23% on pea and rice proteins, respectively. Cyanidin-3-glucoside and cyanidin-3,5-diglucoside had adsorption capacities of 35% and 12% on pea proteins, while on rice proteins, the adsorption capacity was 31% and 8%, respectively. Delphinidin-3-glucoside and delphinidin-3,5-diglucoside had higher affinity toward proteins than cyanidin derivatives. Adsorption capacities were 40% and 30% for pea proteins, and 30% and 24% for rice proteins, respectively. Ellagic acid had a high affinity towards proteins, with 80% and 70% for pea and rice proteins, respectively. The other phenolic acid, i.e., gallic acid, had a lower adsorption capacity and was the only polyphenolic compound with higher affinity towards rice proteins, 58% compared to 51% for pea proteins. Punicalagin, a specific polyphenol in pomegranate juice, had the highest affinity for proteins of all detected polyphenols, with 88% and 80% for pea and rice proteins, respectively.

The results of total polyphenols, proanthocyanidins, and the determination of antioxidant activities of the pomegranate juice and the generated protein microparticles are displayed in Table 1. Pomegranate juice contained 8.48 mg GAE/mL of total polyphenols and 6.34 mg PB2E/mL of proanthocyanidins. Antioxidant activity values for the DPPH, ABTS, FRAP, and CUPRAC protocols were 32.01, 51.30, 52.92, and 53.94 µmol/mL, respectively. The total polyphenol content was approximately 3.8 GAE mg/g on both protein microparticles, while the pea protein microparticles contained a higher amount of proanthocyanidins than rice protein microparticles (2.19 and 1.33 PB2E mg/g, respectively). Consequently, the values of antioxidant activities determined by the applied methods were higher for pea protein microparticles. The antioxidant activities for pea protein microparticles were 18.50, 38.04, 37.33, and 36.92 TE µmol/g, as estimated by the DPPH, ABTS, FRAP, and CUPRAC protocols, while for rice protein microparticles these values were 15.86, 34.19, 34.31, and 35.76 TE µmol/g.

The concentration of individual polyphenols in protein microparticles is presented in Table 2. Two cyanidin and two delphinidin anthocyanin derivatives were determined in protein microparticles. Although the adsorption capacity was higher for delphinidin derivatives, cyanidin derivatives were found in higher concentrations on the microparticles. Cyanidin-3-glucoside and cyanidin-3,5-diglucoside were assessed at concentrations of 135.26 and 132.33 mg/kg on pea protein microparticles, while on rice protein microparticles these concentrations were 129.45 and 119.67 mg/kg, respectively. As previously noted, delphinidin derivatives were determined in lower concentrations; however, they showed a slightly different trend depending on the protein type. Delphinidin-3-glucoside and delphinidin-3,5-diglucoside were assessed at concentrations of 87.94 and 83.34 mg/kg on pea protein microparticles. On rice protein microparticles, delphinidin-3,5-diglucoside was determined in a higher concentration (75.27 mg/kg) than delphinidin-3-glucoside (68.60 mg/kg). Two phenolic acids, ellagic and gallic acids, were also identified, exhibiting different trends depending on the protein type. Ellagic acid was determined in the concentrations of 25.98 and 14.58 mg/kg on pea and rice protein microparticles, respectively. In contrast to ellagic acid, gallic acid was determined in much higher concentrations, 181.92 and 199.61 mg/kg, with a higher concentration on rice protein microparticles. Punicalagin was determined in concentrations of 86.99 and 70.9 mg/kg on pea and rice protein microparticles, respectively.

### 3.2. Inhibition of Colon Cancer Cells

**Figure 2:**
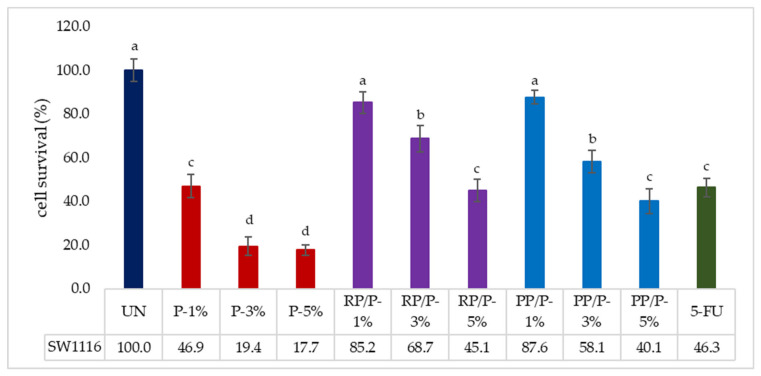
Cell survival of SW1116 (early-stage) colon cancer cells after treatment with pomegranate juice and protein microparticles extracts (UN—untreated cells, P—pomegranate juice, RP/P—rice protein microparticles, PP/P—pea protein microparticles, 1%, 3%, and 5%—amount of juice/extract used for treatment of cancer cells, 5-FU—5-fluorouracil). Values a, b, c, and d represent statistically different results by ANOVA and Fisher’s (LSD) test with *p* < 0.05.

**Figure 3:**
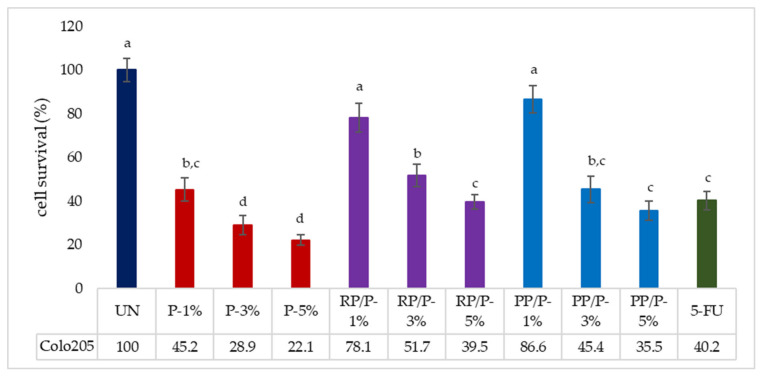
Cell survival of Colo205 (late-stage) colon cancer cells after treatment with pomegranate juice and protein microparticles extracts (UN—untreated cells, P—pomegranate juice, RP/P—rice protein microparticles, PP/P—pea protein microparticles, 1%, 3%, and 5%—amount of juice/extract used for treatment of cancer cells, 5-FU—5-fluorouracil). Values a, b, c, and d represent statistically different results by ANOVA, Fisher’s (LSD) test with *p* < 0.05.

Initially, SW1116 (early-stage) and Colo205 (late-stage) colon cancer cells were treated with pomegranate juice to investigate whether polyphenols in pomegranate juice affect the survival of cancer cells (Figure 2 and Figure 3). To compare the impact of pomegranate juice, results from untreated cancer cells and cells treated with 5-fluorouracil, which served as a positive control (i.e., a drug used in the treatment of colon cancer), are shown. Both types of cancer cells were treated with 1%, 3%, and 5% of pomegranate juice, and inhibition of cancer cell survival was recorded. As the amount of pomegranate juice increased, the survival of SW1116 (early-stage) colon cancer cells decreased to 46.9%, 19.4%, and 17.7%, respectively. Similar results were observed for Colo205 (late-stage) colon cancer cells, where increasing the amount of pomegranate juice reduced cell survival to 45.2%, 28.9%, and 22.1%, respectively.

Both types of colon cancer cells were treated with extracts of pea and rice protein microparticles, which, as previously explained, contained polyphenolic compounds and exhibited antioxidant activity. As with pomegranate juice, colon cancer cells were treated with 1%, 3%, and 5% of protein microparticle extracts. The results of these experiments are shown in Figure 2 and Figure 3. As was expected, since adsorption of polyphenols on protein matrices was not complete, lower inhibition of cancer cells was obtained. Under the defined experimental conditions, extracts from both types of protein microparticles affected the inhibition of both types of colon cancer cells. As the amount of pea protein microparticle extract used for treatment increased, the survival of SW1116 (early-stage) colon cancer cells decreased to 87.6%, 58.1%, and 40.6%, respectively. For Colo205 (late-stage) colon cancer cells, survival decreased to 86.6%, 45.4%, and 35.5%, respectively. It is evident that with 3% and 5% of extract, greater inhibition of Colo205 colon cancer cells was achieved. Rice protein microparticle extracts were also effective in inhibiting the selected colon cancer cells (Figure 3). With increasing amounts of rice protein microparticle extract, the survival of SW1116 colon cancer cells decreased to 85.2%, 68.7%, and 45.1%, respectively. The survival rates for Colo205 colon cancer cells decreased to 78.1%, 51.7%, and 39.5%, respectively. As observed with pea protein microparticles, a similar trend was seen with rice protein microparticles: at 3% and 5% of extract, greater inhibition of Colo205 colon cancer cells was achieved compared to SW1116 cells. A dose-dependent trend in inhibition was observed for both cell lines, supporting the biological relevance of polyphenol concentration in determining antiproliferative efficacy.

The results show that application of 5% of both protein microparticle extracts resulted in the same inhibition of SW1116 cancer cells as 1% pomegranate juice. For Colo205 inhibition, 3% and 5% of both protein microparticle extracts resulted in the same inhibition as 1% pomegranate juice. These findings indicate that polyphenols adsorbed onto protein to form microparticles had an inhibitory effect on selected colon cancer cells, and the results are comparable to those of the standard drug, 5-fluorouracil.

### 3.3. Changes in Protein Structure

**Figure 4:**
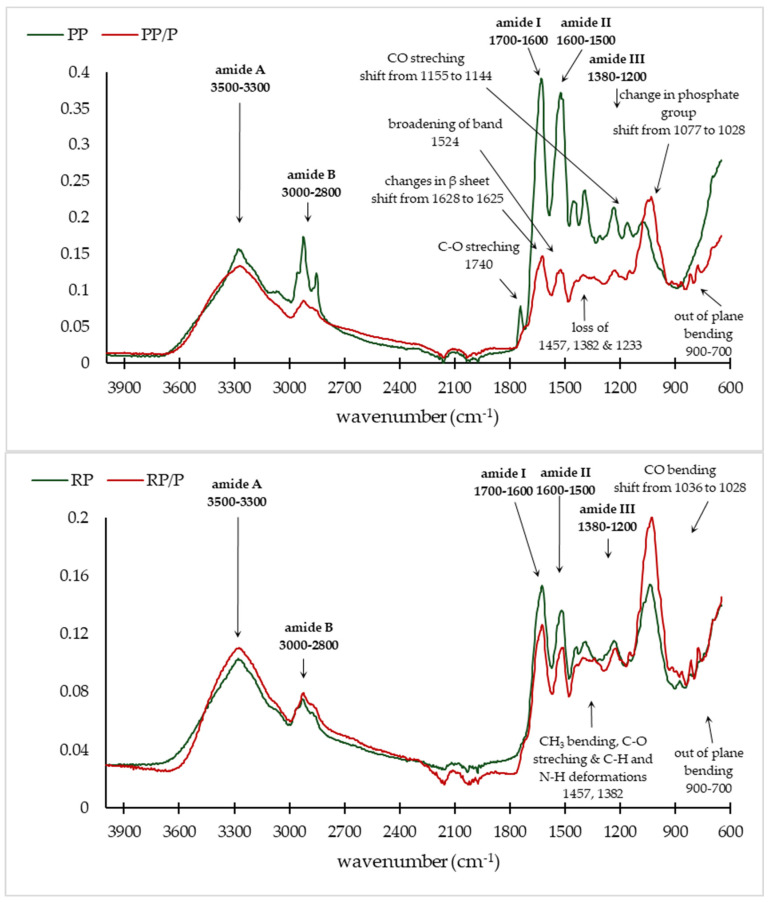
IR spectra of plant protein matrix (PP—pea proteins and RP—rice proteins) and related microparticles generated with pomegranate juice (PP/P—pea protein microparticles and RP/P—rice protein microparticles).

Recorded IR spectra of the plant protein matrices and related microparticles generated with pomegranate juice are shown in Figure 4 to provide information about the alterations of protein structure due to the adsorption of polyphenols. Well-documented characteristic wavelength intervals for protein structures, known as amide bands, are 1700–1600 cm^−1^, 1600–1500 cm^−1^, 1380–1200 cm^−1^, and 3300–3500 cm^−1^. These intervals correspond to specific amide structures: amide I (corresponding to C–O stretching), amide II (corresponding to N–H bending and C–H stretching), amide III (corresponding to N–H in-plane bending coupled with C–N stretching), and amide A structures of proteins, respectively [34,35,36,37]. Protein secondary structures comprise α-helix, β-sheet, β-turn, and random coil, which are associated with the intervals 1658–1650 cm^−1^, 1640–1610 cm^−1^, 1700–1660 cm^−1^, and 1650–1640 cm^−1^, respectively [36]. Both protein matrices were affected by the adsorption of polyphenols. Adsorption of pomegranate polyphenols resulted in changes in the intensity of bands in both proteins. The band in the interval 3100–2980 cm^−1^, which was lost due to adsorption of pomegranate polyphenols on pea proteins, indicates a change in the amide B structure (corresponding to N–H and C–H stretching). Adjacent to this interval, the 3000–2800 cm^−1^ region defines hydrophobic interactions between polyphenols and proteins [38]. In the pea protein structure, significant modification of this interval was observed. The adsorption of polyphenols on rice proteins did not affect those intervals. This indicates different types of interactions.

Unlike rice proteins, pea proteins exhibited a distinctive band at 1740 cm^−1^, which disappeared after adsorption of polyphenols, indicating alterations in C–O stretching. The amide I structure of pea proteins was modified, as shown by a shift in the band from 1628 to 1625 cm^−1^, indicating changes in β-sheet content [36]. Additionally, the amide II structure was modified, evidenced by broadening of the band at 1524 cm^−1^. Further distinctive modifications in the IR spectra were observed at 1457, 1382, and 1233 cm^−1^, as all these bands were lost after polyphenol adsorption. These modifications correspond to asymmetric CH_3_ bending of the methyl groups of proteins, stretching of C–O with deformation of C–H and N–H, and changes in the amide III structure, respectively [39]. There was also a shift in the band at 1155 cm^−1^ in proteins to 1144 cm^−1^ in pea protein aggregates, indicating alternations in C–O stretching vibration. A pronounced shift in the band at 1077 cm^−1^ in pea proteins occurred upon adsorption of polyphenols, shifting to 1028 cm^−1^, indicating changes in the phosphate group [39].

In contrast to pea proteins, no other modifications in the amide I and amide II structures were observed in rice proteins after adsorption of polyphenols, except for changes in band intensity. The same modifications as in pea protein structure occurred at 1457 and 1382 cm^−1^ after the adsorption of polyphenols. These modifications correspond to asymmetric CH_3_ bending of the methyl groups of proteins, stretching of C–O with deformation of C–H and N–H, and changes in the amide III structure, respectively [36]. There was also a shift in the band at 1036 cm^−1^ in rice proteins to 1028 cm^−1^, indicating changes in stretching coupled with C–O bending. Additional modifications in both protein structures happened in the range of 900 cm^−1^ to 700 cm^−1^, which are associated with out-of-plane bending vibrations [39].

## 4. Discussion

Globulin is the major protein fraction in pea proteins (65–85%), followed by albumin and a small portion of prolamin and glutelin. The main types of globulin are legumin and vicilin in a 2:1 ratio. Legumin contains more sulfur-containing amino acids than vicilin; thus, the disulfide bonds cause stabilization. The secondary structure of pea proteins presents a combination of α-helices and β-sheets, contributing to relatively ordered and flexible conformations. Studies have shown that legumin possesses higher structural order and hydrophobicity, whereas vicilin exhibits a more flexible and less compact structure [40]. This type of structure enables the creation of hydrogen bonds, hydrophobic and π-π interactions between polyphenols and free –NH_2_ and –COOH functional groups on side chains [14,41]. Additionally, the availability of aromatic amino acids results in π-π interactions [42]. Glutelins are the dominant fraction in rice proteins, and in comparison to the pea globulin fraction, they differ in their structure and organization. Additionally, rice proteins contain small portions of prolamin, albumin, and globulin. While pea proteins have well-defined oligomeric structures, rice glutelins are characterized by heterogeneous, high-molecular-weight networks. Structurally, they are richer in β-sheet conformations [43]. As in the case of pea proteins, the free side chain functional groups -NH_2_ and –COOH on rice proteins, in addition to the creation of hydrogen bonds with polyphenols, cause electrostatic interactions between compounds [44], while aromatic amino acids enable π-π interactions [45].

These structural differences result in varying affinities for the adsorption of pomegranate polyphenols, while the structures and characteristics of the polyphenols themselves also influence their adsorption onto protein matrices [46]. As in other studies, our data indicated that various types of polyphenols vary in their affinity to adsorb on proteins; thus, punicalagin and phenolic acids had a higher affinity for adsorption to both protein matrices than other polyphenols. The main structural characteristics of polyphenols that define their reactivity and binding capability to proteins are the number and location of hydroxyl groups [47]. Consequently, our data showed the highest adsorption capacity for punicalagin. This may also be related to the structure of anthocyanins, as delphinidin derivatives had a higher adsorption capacity than cyanidin derivatives. However, the adsorption capacities of anthocyanins were significantly lower compared to those of punicalagin and phenolic acids. The complexation mixture contains a large amount of water, creating conditions that initiate diffusion-dependent reactions. Usually, higher proportions of water result in the degradation of unstable compounds such as phenolics, among which anthocyanins are especially sensitive to water. The presence of water increases molecular mobility, which consequently enhances hydrolysis and oxidation reactions. Anthocyanidins, which are even more chemically unstable than anthocyanins, are formed as a result of the hydrolysis of the glycosidic bond in the anthocyanin structure. Often, after this chemical change, the opening of the pyrilium ring happens, which can further lead to the formation of chalcones and brown end-products [48]. In addition, the availability of oxygen accelerates the oxidation rate of anthocyanins. During the complexation of proteins and polyphenols, in addition to the interactions between these compounds and the impact of water, an additional mechanism should be considered for the adsorption of polyphenols on proteins. Most likely, a stacking effect, i.e., interactions between anthocyanins from juice and anthocyanins already bonded to protein, can occur [15,49,50]. Thus, adsorption is a consequence of several different factors.

Many studies were performed with the goal of studying the effect of different proteins and polyphenol structures on their interactions. Comparing adsorption capacities on the same protein matrices, it was determined that rice proteins had a higher adsorption capacity than pea proteins for total polyphenols and proanthocyanidins when chokeberry juice polyphenols were adsorbed on them, while the adsorption capacities for monomeric anthocyanins were similar for both proteins [15].

Gallic acid showed the highest affinity for soy proteins, followed by chlorogenic acid and quercetin. Lower affinity was detected for myricetin, caffeic acid, and kaempferol, with the lowest for apigenin and flavone [47]. Sęczyk et al. [46] compared the affinity of polyphenols for two fractions of white bean proteins: albumin and globulin. They found that chlorogenic and gallic acids had the highest affinity for albumin, while chlorogenic acid had the highest affinity for globulin. Catechin and quercetin showed lower affinity for albumin, while gallic acid, catechin, and quercetin showed lower affinity for globulin. Apigenin and ferulic acid had the lowest affinity for both fractions [46].

The polyphenols profile and their ratio in the complexation mixture greatly affect their binding to proteins. This was demonstrated by examining the affinity of pure compounds and mixtures, such as extracts of green coffee and green tea, for the previously mentioned protein fractions. The experiment demonstrated that catechin and chlorogenic acid from the extracts had a higher affinity than individual pure polyphenols for protein fractions, probably due to the presence of other polyphenols in the extracts. This emphasizes the significance of the impact of other compounds, i.e., boosting or diminishing the affinity of compounds for proteins [46]. A mixture of polyphenols, such as those found in juice, can result in competition between compounds for the same binding site on protein structures, additionally affecting the adsorption of polyphenols onto proteins.

Comparing the results of antioxidant activities of pomegranate juice and protein microparticles, lower values of antioxidant activity were observed for protein microparticles with all methods. This outcome was expected, as polyphenols did not fully adsorb onto proteins and adsorbed onto proteins in varying ratios, as stated in the previous section. To estimate the antioxidant capacities of the samples, four methods were used. Although these methods differ in their mechanisms of action, all of them showed higher antioxidant capacity for pea protein microparticles, which is directly related to the amounts of total polyphenols, proanthocyanidins, and the concentrations of individual polyphenols. The selected methods are based on the reaction of antioxidants donating hydrogen to free radicals or reducing metal ions. The first mechanism involves the ABTS and DPPH methods, i.e., the donation of hydrogen to synthetic ABTS^+^˙ and DPPH˙ radicals. Lower antioxidant capacity was estimated using the DPPH method. Its higher selectivity is related to the structure of polyphenols, specifically the position and number of hydroxyl groups. DPPH˙ free radicals do not react with flavonoids whose B ring contains no hydroxyl groups, and with aromatic acids that have only one group. On the other hand, ABTS^+^˙ free radicals react with hydrophilic and hydrophobic polyphenols, as well as with any hydroxylated aromatics, regardless of their true antioxidant ability [51]. The second mechanism involves the CUPRAC and FRAP methods, that is, the reduction in Cu^2+^ and Fe^3+^ [52,53]. In contrast to the previous two methods, our results showed that both microparticles have the same affinity for the reduction of the investigated metals. One of the most important structural features affecting antioxidant activity is the number and arrangement of hydroxyl and methoxy groups on the phenolic rings. In addition to structural features, it is important to consider that the ratio between components, their concentrations, interactions, dissociation, and ionization play a significant role in the expression of antioxidant capacity [53,54,55,56].

Colon cancer is among the most diagnosed cancers worldwide, with its incidence rising in the younger population. As bioactive compounds have become the focus of numerous studies for the prevention and potential treatment of cancer, and pomegranate juice polyphenols have demonstrated positive effects, we investigated whether pomegranate juice polyphenols adsorbed onto protein microparticles can inhibit early-stage and late-stage colon cancer cells. Through other studies, researchers also observed a positive impact of pomegranate juice and its purified compounds on the inhibition of induction and proliferation of colon cancer cell lines. In most studies, it has been concluded that the main mechanism for the antiproliferative activity of pomegranate juice on colon cancer involved modulation of COX-2 expression. COX-2 expression presents an indication of an inflammatory signaling reaction that leads to the initiation and progression of HT-29 colon cancer cells [57,58,59,60,61]. Moreover, it was observed that pomegranate juice had a greater positive impact on decreasing COX-2 expression than punicalagin. These findings not only emphasize the importance of the presence of bioactive compounds but also of the interactions among them, including flavonoids, phenolic acids, and anthocyanins. This supports the conclusion that isolated individual polyphenols from pomegranate juice may exhibit reduced overall efficacy due to the absence of synergistic or antagonistic interactions with other components [57,58,59,60,61]. In our study, a positive impact, i.e., the inhibition of both types of colon cancer cells, was achieved by treating the cells with pomegranate juice. As discussed previously, not all polyphenols were adsorbed onto proteins to the same extent; however, the positive effect persisted. The slightly reduced antiproliferative effect observed for protein microparticle extracts compared to pomegranate juice may be attributed to the differences in release kinetics and bioavailability of polyphenols from the protein matrix, rather than a loss of bioactivity.

The importance of interactions between compounds is also evident in our research. Both pomegranate juice and extracts of protein microparticles exhibited an antiproliferative effect on SW1116 and Colo205 colon cancer cells, regardless of different adsorption efficiencies on protein microparticles. Sun et al. [62] investigated the impact of punicalagin on three colon cancer cell lines (HT-29, HCT 116, and LoVo), and their results revealed that punicalagin initiated their apoptosis through an increase in the activity of caspase-3. Moreover, it caused suppression of the expression of MMP-9, MMP-2, Snail, and Slug, which are key molecular indicators in cancer progression [62]. Although the literature highlights punicalagin as one of the most important anticancer polyphenols in pomegranate juice, other compounds also play significant roles. Anthocyanins are particularly important in inhibiting the growth of colon cancer cells. They act as inhibitors of proliferation and promoters of apoptosis in HT29 colon cancer cells by enhancing the Bcl-2/Bax ratio and caspase-dependent apoptotic pathways [63]. Their antiproliferative properties are linked to their structure, specifically the number of hydroxyl groups on the B-ring. Anthocyanins with three hydroxyl groups, i.e., delphinidin, demonstrated a greater inhibitory potential (83%) than cyanidin, which has two hydroxyl groups (48%) [64]. Ellagic acid can inhibit colon cancer cells by inducing cell cycle arrest and apoptosis, as demonstrated in HCT-116 colon cancer cells. Regulation of long noncoding RNAs was suggested as a possible mechanism [65], while another author emphasized the initiation of autophagy through the AMPK/mTOR pathway [66]. Gallic acid also inhibited the proliferation of HCT116 and HT29 cells and caused apoptosis by regulating the proportion of cleaved caspase-3/pro-caspase-3 and cleaved caspase-9/pro-caspase-9. Additionally, this phenolic acid reduced the levels of phosphorylated (p)-SRC, p-EGFR, p-AKT, and p-STAT3 [67]. Moreover, it was observed that gallic acid can inhibit the metastatic ability of colon cancer cells, probably due to the suppression of the integrin/FAK axis mediated by miR1247-3p [68]. Although mechanistic studies were not performed in this work, the observed antiproliferative effects are likely associated with the induction of apoptosis, modulation of oxidative stress, and regulation of inflammatory signaling pathways, as previously reported for pomegranate-derived polyphenols.

The scanning of IR spectra and a comparison of protein matrices with corresponding microparticles showed that the adsorption of pomegranate juice polyphenols has occurred and caused changes in protein structure. Interactions between different types of polyphenols and proteins have been demonstrated in other studies, which concluded that these interactions can be a covalent type and/or non-covalent [69,70,71]. Interactions of non-covalent nature involve the creation of hydrogen bonds, hydrophobic associations, van der Waals forces, and electrostatic attraction, with hydrophobic interactions and hydrogen bonds being the most important driving forces between polyphenols and proteins [72]. In a previous study involving the adsorption of polyphenols from chokeberry juice on pea and rice proteins, it was also determined that the adsorption of polyphenols caused alterations in protein structure. Both protein matrices exhibited changes in amide I, amide III, and amide B intervals, while rice proteins additionally showed alterations in the amide I structure, specifically in the β-sheet interval [15].

Another implication of interactions between polyphenols and proteins forming microparticles that must be considered is that these interactions affect the digestibility of both polyphenols and proteins [3,73,74,75,76,77]. The expression of health benefits from these compounds in the body depends on their bioavailability after ingestion and their behavior as they pass through the gastrointestinal tract, which further determines their potential application as functional food ingredients [74,77]. Over the years, it has been shown that complexing polyphenols with proteins efficiently enhances the health-related effects of polyphenols, as they are protected within the protein matrix while passing through the gastrointestinal tract. This prevents the degradation of polyphenols in the gastrointestinal tract, allowing intact, non-degraded compounds to reach the colon, where further catabolism occurs at the gut microbiome level. Thus, protection of polyphenols by the protein matrix is generally necessary for these compounds to be absorbed in the colon and for their positive effects to be manifested [73,74,75]. This is especially important for anthocyanins, since they are, as already mentioned, highly unstable, and it has been proven that their interactions with proteins are an effective way to enhance their bioactivity, with improved bioaccessibility and bioavailability compared to those delivered without a protein carrier [78]. The biological activity of anthocyanins in the body is almost never expressed as their independent action. Commonly, anthocyanins and other flavonoids, as well as anthocyanins and other nonflavonoid phytochemicals, interact to ensure their full bioactive potential [79]. Thus, in our study, these interactions between polyphenols and proteins can protect polyphenols during passage through the gastrointestinal tract and ensure their bioactivity in the body, not only in terms of antioxidant potential but also possible antiproliferative action on colon cancer cells. Regarding polyphenol–protein interactions, they can also enhance the functionality of proteins and affect their digestibility [3,76,77]. During digestion in the gastrointestinal tract, proteins undergo conformational changes and fragmentation caused by digestive enzymes. Complexes between polyphenols and proteins can influence enzyme activity, especially that of proteases. Two major inactivation mechanisms are associated with polyphenols, which are closely related to the type of interactions between these compounds. One mechanism involves a direct alteration of enzyme activity, while the other affects the structure of the enzyme substrate. When non-covalent interactions are involved, polyphenols can be released from the protein structure due to the low pH of the stomach, allowing the free compound to interact with proteases. In the case of covalent interactions, due to their strength, polyphenols remain bound to the protein structure, also altering protease activity. One way this occurs is by changing the spatial conformation of the protein substrate, making it more or less available for enzymatic hydrolysis; another way is by changing the number of available active sites for enzyme binding [3,76,77,80].

## 5. Conclusions

Pea and rice protein microparticles were prepared by complexing the respective protein matrices with pomegranate juice polyphenols. The results showed that the selected proteins adsorbed polyphenols from pomegranate juice during preparation. The adsorption of polyphenols onto the protein matrices depended on the structure and properties of both the proteins and the polyphenols. The polyphenols adsorbed onto the protein matrices retained antioxidant activity and inhibition of colon cancer cell proliferation to a lesser extent, but the results remained sufficiently high. As a future perspective, this study provides a good baseline for further research, especially regarding the in vitro digestion of polyphenols and proteins, stability over time, and the behavior of the formulated microparticles in real food products.

## Figures and Tables

**Table 1:** Total polyphenols (TPC), proanthocyanidins (PC), and antioxidant activities of pomegranate juice and protein microparticles.

Parameters	Pomegranate Juice	PP/P	RP/P
TPC (GAE mg/g)	8.48 ± 0.04 ^a^	3.85 ± 0.06 ^b^	3.79 ± 0.04 ^b^
PC (PB2E mg/g)	6.34 ± 0.06 ^a^	2.19 ± 0.01 ^b^	1.33 ± 0.01 ^c^
DPPH (TE µmol/g)	32.01 ± 0.41 ^a^	18.50 ± 0.24 ^b^	15.86 ± 0.22 ^c^
ABTS (TE µmol/g)	51.30 ± 0.38 ^a^	38.04 ± 0.06 ^b^	34.19 ± 0.10 ^c^
FRAP (TE µmol/g)	52.92 ± 0.50 ^a^	37.33 ± 0.61 ^b^	34.31 ± 0.87 ^c^
CUPRAC (TE µmol/g)	53.94 ± 0.27 ^a^	36.92 ± 0.49 ^b^	35.76 ± 0.37 ^c^

PP/P—pea protein/pomegranate juice microparticles, RP/P—rice protein/pomegranate juice microparticles, GAE—equivalents of gallic acid, PB2E—equivalents of procyanidin B2, TE—equivalents of Trolox. Values a, b, and c represent statistically different results by ANOVA and Fisher’s (LSD) test with *p* < 0.05.

**Table 2:** The concentration of individual polyphenols (mg/kg) in protein microparticles.

Polyphenols	PP/P	RP/P
Cyanidin-3-glucoside	135.26 ± 0.23 ^a^	129.45 ± 0.24 ^b^
Cyanidin-3,5-diglucoside	132.33 ± 0.38 ^a^	119.67 ± 0.05 ^b^
Delphinidin-3-glucoside	87.94 ± 0.32 ^a^	68.60 ± 0.19 ^b^
Delphinidin-3,5-diglucoside	83.34 ± 0.33 ^a^	75.27 ± 0.01 ^b^
Ellagic acid	25.98 ± 0.19 ^a^	14.58 ± 0.10 ^b^
Gallic acid	181.92 ± 0.44 ^b^	199.61 ± 0.71 ^a^
Punicalagin	86.99 ± 0.77 ^a^	70.94 ± 0.88 ^b^

PP/P—pea protein/pomegranate juice microparticles and RP/P—rice protein/pomegranate juice microparticles. Values a and b represent statistically different results by ANOVA and Fisher’s (LSD) test with *p* < 0.05.

## Data Availability

The original contributions presented in this study are included in the article. Further inquiries can be directed to the corresponding authors.
